# The Relation of Syphilis to Tabes

**Published:** 1902-01

**Authors:** 


### EDITORIAL NOTES AND COMMENTS.

The Business office of THE JOURNAL-RECORD is No. 64 Marietta Street.

The Editorial office is 318-319-320 The Prudential.

Address all Business communications to Dr. M. B. Hutchins, Mgr.

Make remittances payable to THE ATLANTA JOURNAL-RECORD OF MEDICINE.

On matters pertaining to the Editorial and Original Communications address Dr. Bernard Wolff, Atlanta.

Reprints of original articles will be furnished at cost price. Requests for the same should always be made *on the manuscript*.

We will present, post-paid, on request, to each contributor of an original article, twenty (20) marked copies of THE JOURNAL-RECORD containing such article.

### THE RELATION OF SYPHILIS TO TABES.

PROF. FOURNIER at the opening lecture of his course at the St. Louis, Paris, expressed his views upon the relation of syphilis to tabes dorsalis. He called attention to the fact that he had advanced the idea of the causal relation of syphilis in this affection in 1875 when the conception was original. He showed that after a period of somewhat contradictory investigations the opinion was to-day absolutely demonstrated to be true. He only reverted to the question for the purpose of presenting some new and important observations. His statistics concerned 1,000 cases of tabes seen by him in hospital and private practice during the last twenty-six years of incessant labor. Among these 1,000 cases syphilis was established 925 times, that is in the proportion of 92 to 100. Such a large number of observations clears the theory of the element of chance. In this series of 1,000 cases there is a more than a morbid coexistence and it must be admitted that syphilis is the principal cause of the tabes. Where it is not established tabes must be ascribed to an overlooked syphilis (acquired, conceptional, hereditary) or to causes still poorly understood (alcoholism, ergotism). Under what conditions does tabes succeed syphilis? It most often follows a benign syphilis; eight times it manifestly succeeded hereditary syphilis. It occurs among individuals predisposed by neurotic heredity, among those who are suffering from the effects of overwork and 93 times out of 100 it appeared in syphilitic who were insufficiently or not at all treated. Tabes occupies the third position in the manifestations of syphilis, coming after cutaneous syphilides and cerebral syphilis. It is incurable. By its incurability and frequency, the prognosis of the affection darkened. The struggle must be unremittingly waged against this plague which with tuberculosis and alcoholism ravages human society.

---

ATTENTION is called to the action of the Macon Medical Society in regard to change in the present laws concerning certain matters that affect the medical profession. We heartily indorse the action of the society and trust that the much needed reforms will be accomplished. There has never been any just reason why the office of coroner should be held by a layman, except for the antiquity of precedent which has long since lost whatever of justification it possessed at its origin. The duties of the office are properly those that come within the province of the medical profession, so that whether the incumbent be lay or professional, the inquest always requires the testimony of a physician, showing his indispensability.

The present method of adjudicating upon the mental state of an individual in the light of developments in psychiatry leaves much to be desired. The plan suggested of examination of the individual by three physicians, with the ordinary as jury, eliminates many of the unsatisfactory features of the system now in vogue. Insanity is often so elusive as to escape even the expert, and how then is it to be passed upon by a jury of laymen? The resolution to require the formula of proprietary medicines to appear on the label is an excellent one. The passage of such a bill would go far toward relieving the State of quackery which is so rampant in it and which deprives the sick poor of so many hard-earned dollars. The allurement of quackery is its secrecy and mystery. If the composition of the nostrums that gull the people was made public, many of the former would go out of business and the latter stay longer on earth.

As to the last recommendation, that dangerous poisons and drugs of common habit addition shall be dispensed only on a physician's prescription, the need of such reform is too obvious to require comment. The law on this point is more honored in the breech than the observance, though there are doubtless many conscientious druggists who will refuse to dispense such materials without complying strictly to the law. There are many others, however, who not only evade the regulation but even pander to the frailties of mankind by building up a traffic in enslaving drugs.

It is a pleasure and a sign of progress to see that the medical profession is endeavoring to take a hand in framing laws that have hitherto been left to lay judgment.

---

THE Atlanta Society of Medicine, at its meeting on December 19, elected the following officers for the ensuing year: Bernard Wolff, M.D., President; L. P. Stephens, M.D., Vice-President; Dr. E. van Goidtsnoven was re-elected treasurer and Dr. Claude A. Smith, secretary. Dr. T. V. Hubbard was elected to the executive committee and Dr. C. F. Benson was elected to fill out the unexpired term on the executive committee of Dr. J. A. Childs, resigned. The society is in excellent condition, the attendance large and interest in the meetings increasing. Permanent quarters for holding the meetings have been secured in the new Carnegie Library. A section in the library has been turned over to the society and there is every prospect of a good library of its own in the near future.

